# Enhancing Surgery Scheduling in Health Care Settings With Metaheuristic Optimization Models: Algorithm Validation Study

**DOI:** 10.2196/57231

**Published:** 2025-02-11

**Authors:** João Lopes, Tiago Guimarães, Júlio Duarte, Manuel Santos

**Affiliations:** 1 ALGORITMI Research Centre, University of Minho, Rua da Universidade, Braga, 4800-058, Portugal, 351 934373667

**Keywords:** health care, surgery scheduling problem, metaheuristic model, model optimization, surgery scheduling, artificial intelligence

## Abstract

**Background:**

Health care is facing many challenges. The recent pandemic has caused a global reflection on how clinical and organizational processes should be organized, which requires the optimization of decision-making among managers and health care professionals to deliver care that is increasingly patient-centered. The efficiency of surgical scheduling is particularly critical, as it affects waiting list management and is susceptible to suboptimal decisions due to its complexity and constraints.

**Objective:**

In this study, in collaboration with one of the leading hospitals in Portugal, Centro Hospitalar e Universitário de Santo António (CHUdSA), a heuristic approach is proposed to optimize the management of the surgical center.

**Methods:**

CHUdSA’s surgical scheduling process was analyzed over a specific period. By testing an optimization approach, the research team was able to prove the potential of artificial intelligence (AI)–based heuristic models in minimizing scheduling penalties—the financial costs incurred by procedures that were not scheduled on time.

**Results:**

The application of this approach demonstrated potential for significant improvements in scheduling efficiency. Notably, the implementation of the hill climbing (HC) and simulated annealing (SA) algorithms stood out in this implementation and minimized the scheduling penalty, scheduling 96.7% (415/429) and 84.4% (362/429) of surgeries, respectively. For the HC algorithm, the penalty score was 0 in the urology, obesity, and pediatric plastic surgery medical specialties. For the SA algorithm, the penalty score was 5100 in urology, 1240 in obesity, and 30 in pediatric plastic surgery. Together, this highlighted the ability of AI-heuristics to optimize the efficiency of this process and allowed for the scheduling of surgeries at closer dates compared to the manual method used by hospital professionals.

**Conclusions:**

Integrating these solutions into the surgical scheduling process increases efficiency and reduces costs. The practical implications are significant. By implementing these AI-driven strategies, hospitals can minimize patient wait times, maximize resource use, and enhance surgical outcomes through improved planning. This development highlights how AI algorithms can effectively adapt to changing health care environments, having a transformative impact.

## Introduction

### Background

The effective delivery of hospital services depends on the efficient execution of several processes and is becoming increasingly reliant on computer resources capable of responding to specific situations [1]. Given the objectives each health care organization must meet and the requisite patient care, a system capable of improving, evaluating, and preventing future situations is crucial [2,3]. Simultaneously, the volume of patient-centered data in health care has been burgeoning. An example of this is illustrated by Jayaratne et al [4], which highlights the data-rich environment of the intensive care unit, where extensive data streams continuously emanate from patient monitoring and observation. These data encompass various facets such as laboratory results, medical prescriptions, therapeutic decisions, clinical observations by health care providers, and others. The richness of these systems depends on the available data, and the health care system has a great scope at this level. In 2018, Feldman et al [5] showcased the great diversity of these systems, categorizing them into groups based on the data’s nature and associating them with relevant areas of interest. This wide range of systems, which includes clinical and organizational data, makes it an area with a lot of potential. Business intelligence systems are crucial for these entities, which require tools capable of organizing data in a more perceptible way [6,7]. The rise of artificial intelligence (AI) has led to a profound reflection on these systems, allowing them to be transformed to integrate future data and provide advice on optimal decision-making that affects organizational management [3,8,9]. Improving the decision-making process requires combining data storage with analytical tools, applications, and methodologies, and it aims to incorporate and find relationships between existing data [10]. This provides real-time access and facilitates the proper analysis of historical and current data, obtaining insights that were not possible before [11,12].

For a long time, one of the most discussed topics in hospital organizations has been the surgical scheduling problem (SSP). Surgical scheduling was worsened by the COVID-19 pandemic, as numerous specialties had to halt their treatments in order to prioritize the treatment of other patients. There have been clear repercussions from this management change, including an increase in the number of patients on waiting lists and a need for managers to find clinically and organizationally effective approaches to reduce them. A study was carried out in collaboration with the Centro Hospitalar e Universitário de Santo António (CHUdSA) to determine whether a metaheuristic approach could be implemented in a hospital organization as a strategy to reduce waiting time.

### Related Work

Room planning is a task that needs to be addressed in many fields, particularly within health care, and it includes planning operating rooms (ORs) for surgeries. Cost containment and reduction have emerged as primary objectives in health care management, with hospital managers and professionals trying to understand each factor contributing to the total cost of delivering better services. ORs are one of the areas that have been gathering considerable attention since they are the most critical cost center and consume a large proportion of the hospital’s total expenses. Consequently, ORs have substantial potential for cost savings, and the SSP has been studied and has generated a variety of approaches and heuristics [13]. Currently, there is a growing trend in adopting computational tools based on optimization methods. As outlined by Cortez [14], optimization methods are divided into 3 main categories: blind search, local search, and population-based search. Blind search assumes the exhaustion of all alternatives, guaranteeing that all solutions are tested. It is only admissible for discrete search spaces and is easy to implement. The major disadvantage of this technique is its feasibility when the search space is continuous or too large. It tends to require more computational effort since the search is performed through a set of candidate solutions rather than a single solution. Local search is the most modern optimization technique and is based on new solutions that are generated from existing ones. Several methods focus on a local neighborhood through a given initial solution and use previous searches to guide the next. Population-based search presents a new approach to optimization algorithms, using a set of candidate solutions instead of one [15].

Studies conducted by the scientific community suggest that metaheuristic optimization models are promising as a potential solution to the problem at hand, although they diverge on the primary factors directly impacting the performance of a surgical schedule. Some studies adopt a more specific approach, such as the one developed by Fügener et al [13], which introduces a variable called planned capacity slack for OR days with the application of the simulated annealing (SA) algorithm to minimize planned slack and maximize OR use. The planned slack aimed to minimize overtime by absorbing the variability of surgery duration. Min and Yih [16] designed a calendar for patients with uncertain surgical operations. The calendar accounted for the uncertainty in the period of surgery and the availability of resources, such as the surgical intensive care unit, using a stochastic approach to minimize the sum of costs directly related to patients and the expected costs associated with overtime work. Visintin et al [17] propose that creating an effective surgical scheduling system requires grouping patients into homogeneous surgical groups, developing an approach solely focused on this constraint and scheduling surgical groups rather than actual patients. Most authors state that the performance of an OR depends mainly on how surgical activities are scheduled. This perspective is reflected in the study conducted by Su et al [18], which proposed a self-organizing map-based optimization (SOMO) approach to solve the SSP. Banditori et al [19] also introduced a mixed integer programming model, assuming that a hospital’s waiting list cases could be categorized into homogeneous surgical groups based on their anticipated resources. Agnetis et al [20] adopted a surgical scheduling model that was updated on a weekly basis, allocating various specialties to available sessions and considering only patients immediately eligible for surgery, and patients were selected from a waiting list based on parameters such as surgery duration and waiting time. More recently, a similar approach by Brit et al [21] considered multiple factors (including surgeons, equipment, ORs, and recovery ward beds) in their optimization strategy to meet waiting time objectives by maximizing OR use. Tyagi et al [22] explored various models and techniques used for scheduling, emphasizing the importance of strategic (long-term), tactical (medium-term), and operational (short-term) scheduling levels and demonstrating that daily planning and scheduling using detailed procedural times and sophisticated algorithms substantially enhanced OR use. Wang et al [23] proposed a fuzzy model, integrated with a hybrid genetic algorithm for optimization, to address variability and unpredictability in scheduling processes. The model effectively balanced the costs and benefits associated with using an overflow strategy, where patients were assigned to undesignated departments to better manage capacity.

Despite the varied development approaches and the implementations of algorithms, different perspectives have emerged regarding the timescale and surrounding constraints, highlighting the absence of standard approaches to the SSP that conclusively prove effectiveness compared to current hospital management practices. There is a scarcity of accurate proposals to inform the establishment of standard rules and guidelines to manage surgery scheduling without affecting the organizational policies of different surgical specialties. Therefore, this study aimed at demonstrating the effectiveness of a selected set of optimization algorithms, customized to time and resource allocation problems, in addressing common scheduling constraints across various specialties. The primary goal is to provide a more comprehensive solution applicable to different health care organizations.

## Methods

### Ethical Considerations

This study was based exclusively on anonymous data provided by the organization involved in this research and did not involve sensitive personal information. Therefore, ethics board review in accordance with the General Data Protection Regulation (GDPR) was not required.

### Study Design

**Figure 1.**
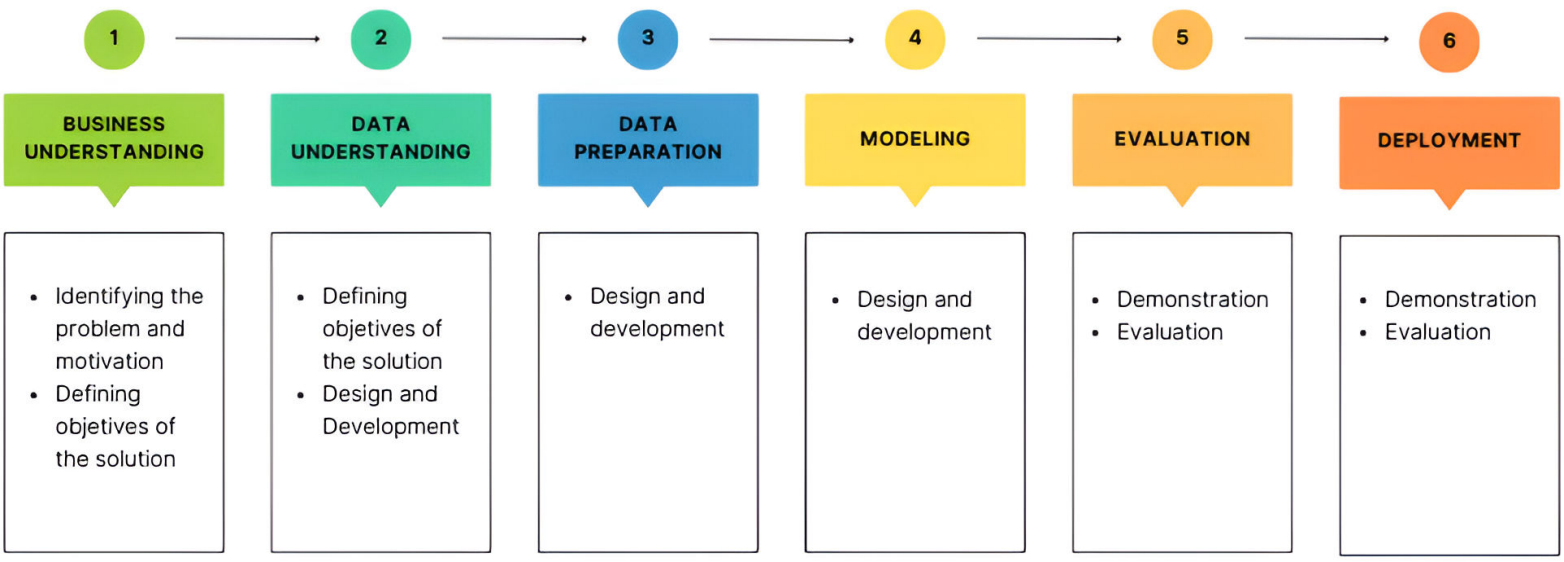
Crossover of the design science research (DSR) and cross-industry standard process for data mining (CRISP-DM) methodologies.

An optimization model–based approach was developed to demonstrate the application of heuristic algorithms. Our analysis focuses on optimizing the temporal allocation of surgeries, depending on the date they were placed on the waiting list and their priority. For this, two methodologies were followed: the design science research (DSR) and the cross-industry standard process for data mining (CRISP-DM). DSR consisted of 6 phases: identification of the problem and motivation, definition of objectives of the solution, design and development, demonstration, evaluation, and communication. These phases provided guidelines for a research project [24]. To put DSR in action, it was necessary to use a practical methodology for data mining projects. The CRISP-DM method provided a global perspective on the life cycle of a data mining project, and it comprised the following 6 stages: business understanding, data understanding, data preparation, modeling, evaluation, and deployment [25]. There were dependencies between the stages, and they did not have a rigid structure [25]. Since both methodologies were used concurrently, the relationship between them throughout the project phases was described (Figure 1).

### Problem Statement

Hospital administrators attempt to reduce costs while providing the best care possible for their patients. To achieve this aim, they take into account a number of factors that directly affect the quality of surgery scheduling. These factors include the number of professionals available for each day, the specialty related to each type of diagnosis, the availability of slots, and the ability to perform new admissions, with an aim of continuously reducing waiting time. Under this perspective, the surgical area of a hospital was the main optimization target for the development of a metaheuristic method. Each surgical area of a hospital was made up of *S* ORs, a finite *H* number of days, and a group of *N* patients who were waiting for a surgical appointment. Thus, the algorithm allocated patients to available ORs and determined the sequence of surgeries to be performed. A surgery time *ST* was assigned to each patient in *{1, ...N}*. This time included the period of a procedure as well as extra time for preoperative cleaning and preparation. Depending on the type of study, an organization may establish different restrictions that directly influence how the algorithm generates solutions, which are referred to as hard (primary objectives) and soft (secondary objectives) constraints.

Finally, the goal was to define a global optimization strategy for more than one specialty. Hard constraints included a maximum limit for the number of candidates assigned to a vacancy, room capacity constraints, and time constraints, so that the algorithms always considered shifts that were available in certain time blocks. Simultaneously, this included reducing the number of patients whose surgeries exceeded the time limit of the guaranteed maximum response time. Additionally, it was possible that not all ORs were open every day, with some ORs only open during specific times. Each shift therefore indicated the day and the OR. The following points were design factors for this approach:

1. Each specialty was assigned one, more than one, or no OR. These assignments were decided in the stage before the block schedule.
2. Patient priority was defined based on medical and waiting time factors, always considering a prioritized patient list for surgeries.
3. Each surgical specialty managed its patients independently.
4. The hospital contained a specific set of ORs, which each one being unique and adapted for certain types of interventions.
5. A surgery that was programmed after its deadline earned a penalty depending on the priority.
6. Each surgery had information regarding the time required to clean the room and prepare it for next surgery.
7. A patient could not be operated on more than once in the same scheduling period.

For this algorithm, all surgeons were able to be assigned to a surgery. Daily availability plans of the CHUdSA were also considered so that a specific surgery allocation respected the existing resources for each day, namely the number of ORs and available medical professionals. The following sections explain the heuristic approaches.

### Data Understanding and Preparation

The surgery data analyzed was considered event-based. Each surgery undergone by a patient represented an event associated with a medical specialty at a specific time, comprising the execution of all requisite procedures. Additionally, data containing information related to the time blocks available in each specialty were used. The data were derived from medical specialties previously selected by the CHUdSA interlocutors, including a scheduling process carried out in 2019. This time frame was chosen since the hospital administration did not take pandemic constraints into consideration, making it possible to examine a scheduling procedure under standard circumstances without any unusual restrictions.

### Proposed Metaheuristic Algorithms

#### Overview of Metaheuristic Algorithms

Metaheuristic algorithms are search procedures designed to find better solutions to an optimization problem that is considered complex to solve [26]. They are categorized according to how they operate in the search space and how new solutions will be discovered [14] such as nature-inspired versus non–nature-inspired, population-based versus local search, dynamic versus static objective functions, and one versus multiple neighborhood structures [27]. The typical structure is based on 3 main code sections: (1) initial solution, representing the first structure of the problem and ensuring an initial guess, often called a “starting point” for the algorithm; (2) evaluation function, which analyzes a possible solution in the context of the problem, comparing different solutions and providing a ranking or a quality measure score; and (3) objective function, which is composed of the implementation of different optimization algorithms [28].

#### Hill Climbing

Hill climbing (HC) is a local optimization algorithm that climbs a hill until a local optimum with the goal of maximization. The method iteratively searches for new solutions inside the existing solution’s neighborhood, adopting a new solution if it is better than the previous one [29]. The purpose is to discover an improvement by running an extensive search within the defined neighborhood borders and the ability of the algorithm to produce successful results is determined entirely by the initial solution [14,15].

#### Simulated Annealing

Metropolis et al [30] designed SA, which is primarily inspired by a cooling operation in heating-based metallurgy. SA begins with a randomly generated initial solution and a high-temperature *T*. During the cooling phase, the algorithm will converge to an estimated solution, moving away from the local optimum to locate the near-optimal solution, and the method will grow more accurate with each iteration to obtain a better solution [14]. Another solution will be randomly created near the initial solution and the difference in function values is calculated as follows:


(1) $$∆=f\left(xn\right)-f\left(xc\right)$$

If the Δ is smaller for the new solution, the new solution automatically becomes the current solution from which the search will continue [26].

#### Particle Swarm

In 1995, Kennedy and Eberhart conducted a study on the social behavior of a group of animals and concluded that being in a group increases one’s chances of surviving [14]. This is corroborated by the fact that species share information, which increases the probability of finding the optimal hunting location. The particle swarm (PS) algorithm operates on this conceptual foundation, attempting to identify the best solution in a high-dimensional space. It aims at either maximizing profits or limiting losses. There can be multiple local maximums and minimums in a function but only one global maximum and/or minimum. In summary, PS is a population-based algorithm and can be defined by the direct and indirect interactions between distinct sets of information that affect the results obtained, consequently preserving an organized pattern among the dataset [14].

### Modeling the Initial Solution

By assigning one slot to each input operation, the initial solution was obtained in random or sequential slots using the list of specialist surgeries. Depending on the number of slots and surgeries, the bottom and upper parameters were defined as the highest and lowest values for each dimension. The initial solution was obtained by assigning surgeries in available slots for a specific specialty and was implemented by the first fit approach [31,32]. The challenges with this initial method were respecting the time limits connected with each surgery and the turnovers associated with the addition of surgeries to a particular slot. It was also decided that the production of the first solution should include all potential constraints. The graphical representation of how the first solution was generated is included in Multimedia Appendix 1.

The performance was evaluated using a function designed for this purpose. Each solution examined the assigned surgeries by specifying a total solution penalty (*pt*)*,* calculated using the sum of each penalty *p* earned in a surgery, as shown mathematically below:


(2) $$pt=\sum_{i=1}^{i}pi$$

Each penalty was calculated by multiplying the number of days that the surgery was overdue by each deadline (*ds*) and the priority associated with that same surgery (*pr*), presented in the following equation:


(3) $$p=f\left(ds\right)-f(pr)$$

### Modeling the Objective Solution

#### HC Implementation

Different algorithms were used to model the objective solution, which through several iterations, search for a better solution than the existing one. For each iteration, a total penalty was given to the surgeries, making comparisons with other solutions. The iterations ended at the defined limit, returning the best solution. HC implementation was retrieved and adapted from Cortez [14] and could be represented by the following function: *hclimbing* (*par*, *fn*, *change*, *control*, *type*). The input variables were presented as follows:

1. The initial solution (*par*) was obtained by allocating the surgeries to available slots (explained in previous section).
2. The evaluation function (*fn*) evaluated the total penalty of the allocated surgeries.
3. A change function (*change*) was responsible for generating the next candidate, creating minor disturbances in the initial solution by swapping surgeries between different slots and evaluating if this was profitable.
4. The variable *control* was a list that indicated the number of interactions to execute and the information to collect throughout the solution.
5. A last variable (*type*) indicated the main goal of minimization.

#### SA Implementation

SA implementation was also adapted from Cortez [14]. It used a variable temperature as opposed to HC, which chose a fixed value for this control parameter. Starting at a high temperature, the method gradually lowered the control parameter until it reached the desired minimum value or a predetermined number of iterations. The following function represents the SA implementation: *simulated_annealing* (*func*, *par*, *niter*, *step*). The input variables were presented as follows:

1. The evaluation function (*func*) that computed the total penalty, similar to HC.
2. The initial solution (*par*), also similar to HC.
3. Maximum number of iterations (*niter*).
4. Parameter to control the cooling speed of the model (*step*).

#### PS Implementation

The implementation of PS followed a different perspective from that presented in the other two models. It is an algorithm that seeks to efficiently search within a specified boundary, using the iterations between particles to find the best solution possible [14,33]. The implementation of this method was obtained by the *psoptim* method from the *pso* package [34]. The function described has 6 input parameters: *psoptim* (*par*, *fn*, *lowe*r, *upper*, *control*, *eval_func*). These were represented by the following:

1. Vector containing the initial list of surgeries to schedule (*par*).
2. Penalty minimisation function (*fn*).
3. Lower bounds on the variables, belonging to the minimum scheduling shift (*lower*).
4. Upper bounds on the variables belonging to the maximum scheduling shift (*upper*).
5. List of control variables (*control*) belonging to the best solution found, number of interactions, swarm size, and continuous trace of solutions found.
6. Evaluation function to compute the total penalty (*eval_func*), similar to the other methods.

## Results

The number of surgeries performed by each specialty and the number of surgeries performed after the established deadline could be used by the CHUdSA to categorize its OR management. Such variables determined the total penalty of the hospital, which translated into costs that the hospital would have to assume. Table 1 represents a general analysis of the existing data, considering the specialties chosen for this study, to understand the relationship between the number of surgeries being allocated and the number of existing slots for each specialty. For the specialties that were chosen, the number of surgeries to be allocated was higher than the number of existing slots. In this case, optimizing ORs was required to handle the greatest number of surgeries with available resources.

**Table 1.** General analysis between number of surgeries in each specialty with available operating rooms.

Medical specialties	Number of surgeries	Number of time slots
Obesity	198	122
Urology	133	89
Pediatric plastic surgery	98	45

The first approach was carried out to understand which algorithms obtained the best performance and considered the optimization objective, which was the minimization of the penalty and the maximization of the number of surgeries scheduled within the deadline. Table 2 summarizes the values obtained in implementing the algorithms described above.

**Table 2.** General analysis of optimization algorithms performance.

Algorithms	Penalty score by medical specialty		
	Obesity (n=198)	Urology (n=133)	Pediatric plastic surgery (n=98)
Hill climbing	0	0	0
Simulated annealing	5100	1240	30
Particle swarm	42,702	30,000	53,108

Since this initial approach considered a first custom solution with the defined scheduling rules, the local search algorithms obtained better scheduling performances. Consequently, a deeper examination of the HC and SA algorithms was done. The implementation of these algorithms led to a set of results presented in Tables3  4. These values compared the performance of the algorithms in response to SSP.

**Table 3.** Measure of the impact of the hill climbing (HC) algorithm.

Metrics by specialty	Penalty score	Surgeries without penalty, n (%)	Surgeries with penalty, n (%)	Unscheduled surgeries, n (%)
Obesity (n=198)				
CHUdSA^a^ management	643,550	4 (2)	194 (98)	0 (0)
HC optimization	0	190 (96)	0 (0)	8 (4)
Urology (n=133)				
CHUdSA management	37,030	91 (68.4)	42 (31.6)	0 (0)
HC optimization	0	127 (95.5)	0 (0)	6 (4.5)
Pediatric plastic surgery (n=98)				
CHUdSA management	14,760	81 (81)	17 (17)	0 (0)
HC optimization	0	98 (100)	0 (0)	0 (0)

a CHUdSA: Centro Hospitalar e Universitário de Santo António.

**Table 4.** Measure of the impact of the simulated annealing (SA) algorithm.

Metrics by specialty	Penalty score	Surgeries without penalty, n (%)	Surgeries with penalty, n (%)	Unscheduled surgeries, n (%)
Obesity (n=198)				
CHUdSA^a^ management	643,550	4 (2)	194 (98)	0 (0)
SA optimization	5100	164 (82.8)	1 (0.5)	33 (16.7)
Urology (n=133)				
CHUdSA management	37,030	91 (68.4)	42 (31.6)	0 (0)
SA optimization	1240	128 (96.2)	4 (3)	1 (0.8)
Pediatric plastic surgery (n=98)				
CHUdSA management	14,760	81 (83)	17 (17)	0 (0)
SA optimization	30	64 (65)	1 (1)	33 (34)

a CHUdSA: Centro Hospitalar e Universitário de Santo António.

## Discussion

### Overall Results

**Figure 2.**
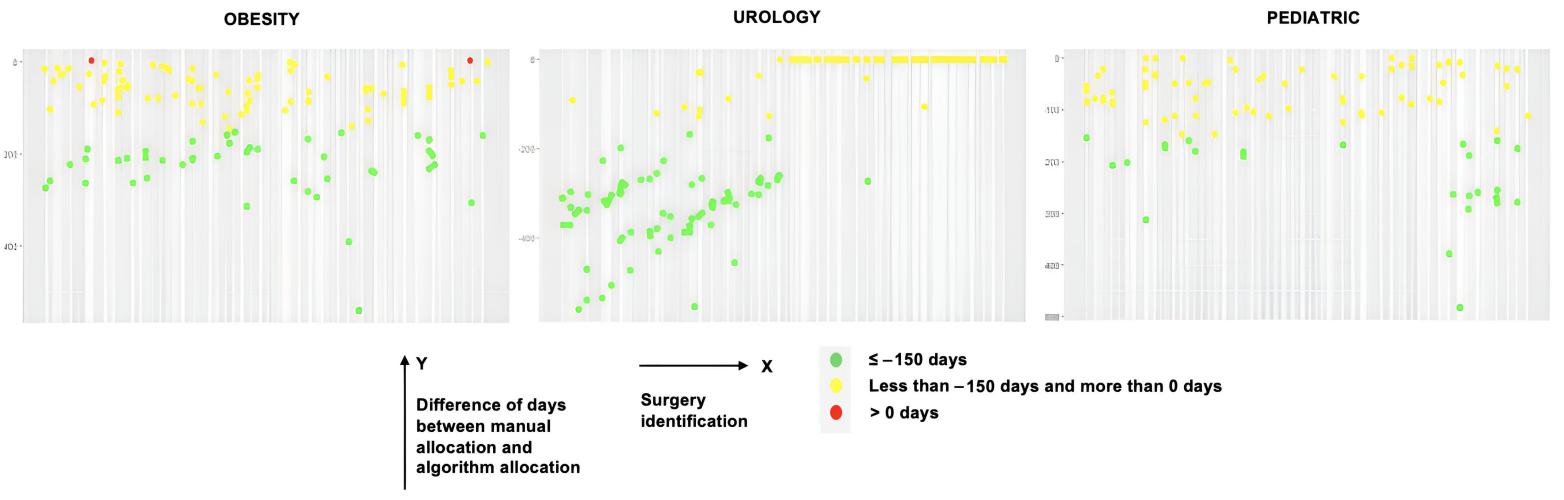
Hill climbing evaluation and overall performance by surgeries.

Through the implementation of these algorithms, compared to CHUdSA’s manual scheduling process, several results were found. First, the penalties for the optimization algorithms developed were lower than the penalty of scheduling done by CHUdSA professionals, and in certain situations, there were no penalties for the algorithms. Therefore, both local optimization algorithms can provide improvements to the administration and organization of ORs. Second, the PS algorithm was not a viable solution to the SSP, since its ability to minimize the scheduling penalty was much lower than the HC and SA algorithms. It was evident that local search algorithms produced better solutions on all existing criteria, compared to the population-based search algorithm. Third, Figures2  3 represent the evaluation of scheduling over time for each surgery, providing an understanding of whether the respective algorithm scheduled a surgery for before or after the day it was performed in the CHUdSA. The majority of surgeries could have been scheduled and carried out days earlier for either of the local HC and SA optimization algorithms. Fourth, the number of surgeries that remained to be scheduled occasionally exceeded the number that the CHUdSA professionals had anticipated. Furthermore, some surgeries could not be scheduled in this optimization process because their minimum execution time exceeded the maximum duration of an existing shift. These surgeries were always handled as exceptional cases at the recommendation of CHUdSA professionals. Therefore, from a management perspective, the professionals personally should schedule the surgeries in accordance with specific internal protocols.

**Figure 3.**
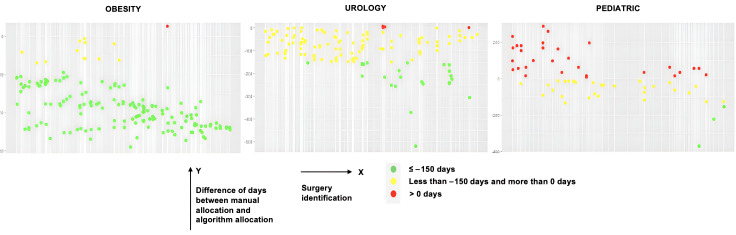
Simulated annealing evaluation and overall performance by surgeries.

### Key Findings

Figures2  3 highlight that it is possible to more effectively allocate surgeries within the same time frame despite using the same resources. Leveraging the specification of a set of global variables for enhancing the scheduling process, 4 significant conclusions can be drawn. First, scheduling was greatly improved when the initial solution was modeled using a surgery allocation algorithm that took waiting list longevity and priority into account. The final penalty was lowered, demonstrating the potential to enhance surgical management within the constraints of time and space. Second, the HC algorithm had the best performance, with the SA algorithm producing similar results. However, the PS algorithm was not able to improve surgery allocation and occasionally arranged surgeries worse in terms of time and space. For these reasons, it was discarded.

Third, the HC and SA algorithms were particularly noteworthy since they arrived at the ideal number of iterations after 100 iterations. Thus, these algorithms could yield significantly better planning than manual hospital allocation with a runtime of approximately 30 seconds. These results also supported the exclusion of the PS algorithm for future implementations, as it was a more computationally demanding model. Fourth, the application of AI-based heuristics resulted in a notable enhancement in the quantity of surgeries allocated. Therefore, there is potential for improving OR management with a system capable of maximizing the scheduling procedure for each speciality, demonstrating how the scheduling solutions assigned by the HC and SA algorithms differ significantly in terms of space and time. In terms of a decision support system, it may be best to offer the user a variety of scheduling solutions based on these implemented optimization models, even though it is not necessary to determine which is the better solution. As a result, the user can select the planning that best fits the group of surgeries that require scheduling.

### Comparison With Prior Work

Based on the development and use of optimization algorithms, the proposed research presents an innovative approach for scheduling surgeries from waiting lists. It is crucial to emphasize the primary objective of reducing costs, considering a variety of factors involving different medical specializations. In contrast to prior studies, the research by Fügener et al [13] focuses on the integration of HC and SA to optimize the use of ORs. Min and Yih’s study [16] concentrates on a stochastic surgical calendar tailored for patients with uncertain surgical needs. Similarly, Banditori et al [19] categorized waiting list cases into homogeneous groups, offering a more adaptable solution for diverse medical specialties. However, because the criteria used to make decisions vary among medical specializations, research so far has never produced conclusive and sufficient evidence to support their widespread use in health care organizations. This work stands out from other studies since it uses global and specific variables that are readily applied to any medical specialty without compromising the quality of surgical scheduling, a key factor in reducing operating costs in hospitals. By diverging from previous models and overcoming their limitations, this study provides a more precise and effective solution aimed at maximizing the performance of ORs, benefiting both the patients and the health care professionals involved in the surgical process.

### Conclusions

The study of allocation and scheduling problems is considered complex. When it comes to health care, the responsibility to create an effective solution is even more significant since the priority must always be the care that is provided to patients, while also being aware of the existing resources. The approach developed in this study not only provides a solution to this scheduling problem but also conceives a generic adoption for any health care organization and for a considerable number of medical specialties.

Considering a general constraints model for any health care organization and considering the same constraints for the generation of the initial solution, the implementation of an automatic allocation algorithm proves the ability to find better solutions for surgery scheduling. The HC and SA algorithms demonstrate the capacity to improve the use of ORs and consider, as a reference, the scheduling limit without accumulating penalties. The PS algorithm proved that it needs a greater computational effort. The fact that it does not use an initial solution, according to previously defined programming rules, can explain its unclear results.

Taking into consideration all of the limitations of scheduling and the high level of organizational complexity, this study’s approach can be considered as a possible solution to the SSP as it contributes to the organization of surgeries based on time and cost control, which are crucial to optimize operating costs. HC and SA show extremely satisfactory results, decreasing the number of surgeries with penalty (ie, surgeries with a scheduled date higher than the deadline date). Additionally, these models provide a near optimal solution, reaching a stabilization point after 100 iterations, since the initial solution had already produced very satisfactory results. In addition, from a total of 429 surgeries and considering all specialities, the HC algorithm managed to schedule 415 (96.7%) surgeries, and the SA algorithm scheduled a total of 362 (84.4%) surgeries. The majority of these surgeries were special cases where their duration exceeded the maximum time available each day. In some cases, the number of surgeries not scheduled by the algorithms was higher than the scheduling performed by the hospital, although this is not considered a negative point of this solution.

The proposed application has addressed multiple challenges, offering a scalable solution across diverse organizational frameworks. However, to deepen the utility and precision of our approach, several topics about limitations and future work have been identified. First, testing this study in other health care organizations will be a crucial step to understand if our approach works in different contexts. In addition, it is necessary to improve the accuracy of each estimated surgery time to increase the reliability of the schedule. In this study, an interquartile mean was used to associate each type of surgery through time history, but a more accurate method is needed for an optimal accuracy. Machine learning models could be used to predict the times of each surgery for greater reliability, which is a crucial factor in the allocation of surgeries.

Second, it is also possible to consider more constraints, including subspecialties, in this mapping. This is considered a significant step since the division of ORs includes an allocation to each subspecialty and requires a complete mapping between specialties and the *International Classification of Diseases, Tenth Revision* (*ICD-10*) codes. Third, a deeper study on the nullification of the scheduling penalty may be considered. While a hospital would like to pay the least amount related to surgeries scheduled after the deadline, it may be pertinent to identify whether a proposal with a higher penalty will better serve the scheduling interests. Fourth, another even more complex proposal is to condition the surgical mapping by considering the human resources available for each period. While this is not possible in the current context considering the available data, an annual organization of medical professionals (surgeons, nurses, anesthesiologists) in real time is necessary. Last, improving the efficiency of the optimization method by exploring more models and their configurations is another direction to consider in future research.

The results of this study are summarized below, highlighting the key messages and anesthesiologists that underline the transformative potential of this approach in health care scheduling. First, the integration of AI-based heuristic algorithms improves the efficiency of surgical scheduling, leading to a reduction in patient waiting times. Second, the HC and SA algorithms have demonstrated a higher performance than the scheduling performed by CHUdSA, and either model make it possible to reduce costs in the scheduling process by reducing the penalties associated with surgeries scheduled beyond the deadline. Third, the models developed do not compromise scalability and adaptability, as they can be adapted to various contexts and medical specialties, and a generalized implementation is possible by adding or removing restrictions. Last, the potential of a system that is capable of integrating these models in any organization is proven, optimizing the inherent management processes and consequently the health care provided to patients.

Adopting this approach following these research directions promises to further refine the metaheuristic optimization models for surgery scheduling, ultimately seeking more optimized and agile health care systems that are patient-centered and cost-effective.

## Supplementary material

10.2196/57231 Multimedia Appendix 1 Structure for the initial solution, according to the first fit method.

## Abbreviations

AI: artificial intelligence
CHUdSA: Centro Hospitalar e Universitário de Santo António
CRISP-DM: cross-industry standard process for data mining
DSR: design science research
GDPR: General Data Protection Regulation
HC: hill climbing
*ICD-10*: *International Classification of Diseases, Tenth Revision*
OR: operating room
PS: particle swarm
SA: simulated annealing
SOMO: self-organizing map-based optimization
SSP: surgical scheduling problem
