# Supplementary figures and images for: Enhancing Surgery Scheduling in Health Care Settings With Metaheuristic Optimization Models: Algorithm Validation Study
Source: JMIR Med Inform. 2025 Feb 11;13:e57231. doi: 10.2196/57231 (PMC11840878; doi:10.2196/57231)

## Multimedia Appendix 1


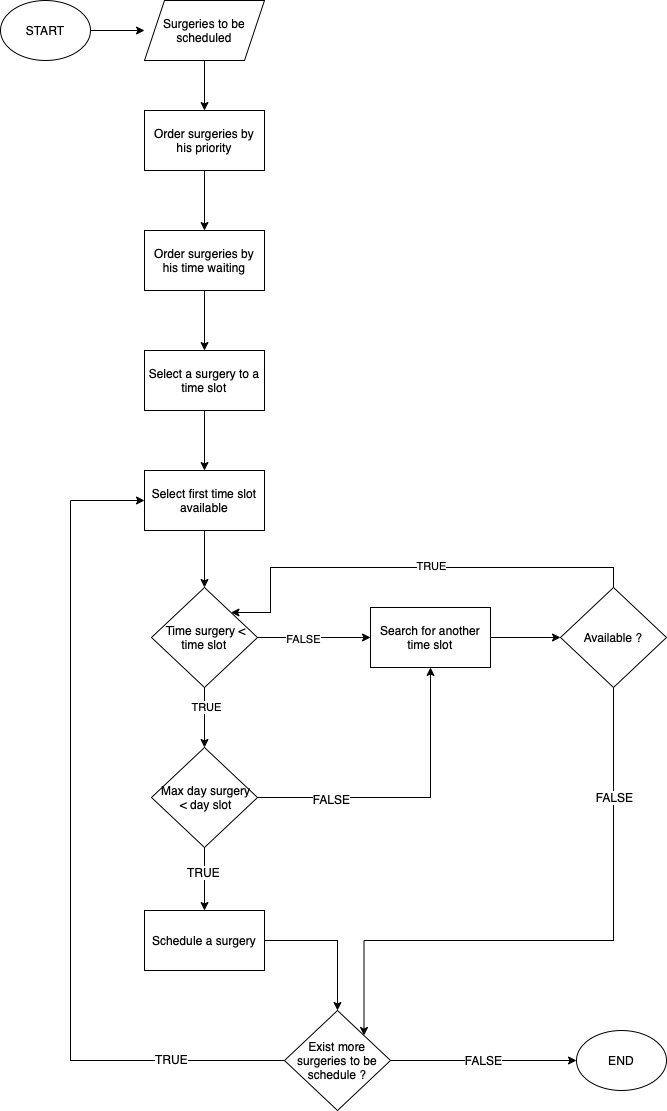


Figure 7: Structure for Initial Solution, according to first fit method.

Supplement: Multimedia Appendix 1 [file medinform-v13-e57231-s001.docx]
